# Early Ongoing Speciation of *Ogataea uvarum Sp. Nov.* Within the Grape Ecosystem Revealed by the Internal Variability Among the rDNA Operon Repeats

**DOI:** 10.3389/fmicb.2018.01687

**Published:** 2018-08-03

**Authors:** Luca Roscini, Mariana Tristezza, Laura Corte, Claudia Colabella, Carla Perrotta, Patrizia Rampino, Vincent Robert, Duong Vu, Gianluigi Cardinali, Francesco Grieco

**Affiliations:** ^1^Department of Pharmaceutical Sciences – Microbiology, University of Perugia, Perugia, Italy; ^2^Institute of Sciences of Food Production (ISPA), National Research Council (CNR), Lecce, Italy; ^3^Department of Biological and Environmental Sciences and Technologies, University of Salento, Lecce, Italy; ^4^Bioinformatics Unit, Westerdijk Fungal Biodiversity Institute, Utrecht, Netherlands; ^5^Centre of Excellence on Nanostructured Innovative Materials (CEMIN), Department of Chemistry Biology and Biotechnology, University of Perugia, Perugia, Italy

**Keywords:** ribosomal DNA, wine yeast, grape ecosystem, internal variability, yeast, concerted evolution

## Abstract

A yeast strain was isolated during a study on vineyard-associated yeast strains from Apulia in Southern Italy. ITS and LSU D1/D2 rDNA sequences showed this strain not to belong to any known species and was described as the type strain of *Ogataea uvarum sp. nov.*, a close relative of *O. philodendri*. Several secondary peaks appeared in the sequences, suggesting internal heterogeneity among the copies of the rDNA. This hypothesis was tested by sequencing single clones of the marker region. The analyses showed different levels of variability throughout the operon with differences between the rRNA encoding genes and the internally transcribed regions. *O. uvarum* and *O. philodendri* share high frequency variants, i.e., variants frequently found in many clones, whereas there is a large variability of the low frequency polymorphisms, suggesting that the mechanism of homogenization is more active with the former than with the latter type of variation. These findings indicate that low frequency variants are detected in Sanger sequencing as secondary peaks whereas in Next Generation Sequencing (NGS) of metagenomics DNA would lead to an overestimate of the alpha diversity. For the first time in our knowledge, this investigation shed light on the variation of the copy number of the rDNA cistron during the yeast speciation process. These polymorphisms can be used to investigate on the processes occurring in these taxonomic markers during the separation of fungal species, it being a genetic process highly frequent in the complex microbial ecosystem existing in grape, must and wine.

## Introduction

The ribosomal RNA encoding region (rDNA) is widely recognized as useful for both phylogenetics and species identification. ITS (Internal Transcribed Spacer) has been proposed as a universal “barcode" for fungi, after a multi-laboratory work (Schoch et al., 2012) and a massive bioinformatics analysis of candidate *loci* (Robert et al., 2011).

This marker increases the possibility already offered by the D1/D2 domain of the Large Subunit (LSU) of the rRNA encoding DNA, previously proposed as species marker sequence (Kurtzman and Robnett, 1998). The rDNA genes are arranged in a large operon with over 100 tandem repeated copies per genome, which have been demonstrated to be somehow not homologous (Kurtzman and Robnett, 1998; Korabecna, 2007; James et al., 2009). This heterogeneity is likely to be due to the presence of different nucleotides in the same position of different copies (Henry et al., 2000).

Even though this genetic feature is sometimes used as the basis for species discrimination, a considerable problem in using this barcode can often be found in the internal variations among copies, which have already been detected for a number of fungal species (Kårén et al., 1997; Aanen et al., 2001; Smith et al., 2007) as well as for other eukaryotes (Gong et al., 2013). This evidence makes it fundamental to study a range of ITS region variants among different species (Horton, 2002; Nilsson et al., 2008). The question goes beyond the interest in fungi because it involves various phylogenetic groupings, e.g., prokaryotes (Stewart and Cavanaugh, 2007), dinoflagellates (Thornhill et al., 2007), mycetes (Connell et al., 2010; Huang et al., 2010; Santos et al., 2010), and different animals (Wörheide et al., 2004; Sánchez and Dorado, 2008; Elderkin, 2009).

Internal heterogeneity is considered a transient situation that will be fixed by concerted evolution, which will homogenize all copies to the most predominant one (West et al., 2014). This model would predict that the intra-genomic heterogeneity is higher in newly formed species and decreases rapidly (Kobayashi, 2011), thus providing additional phylogenetic information on the history of the strains and of the taxa (West et al., 2014).

Concerted evolution is supposed to homogenize the tandem repeats via gene conversion during meiosis (Naidoo et al., 2013). However, the evidence that this phenomenon is also present in non-sexual fungi implies that either the homogenization occurred in the early-stage of the species life in which they were still able to sporulate, thus having a high efficient gene conversion, or via the far less frequent mitotic recombination.

During a large-scale study on vineyard-associated yeast strains from Apulia (Southern Italy), we isolated a strain from “Primitivo” grape berries, here described as a new yeast species of the genus *Ogataea*. This yeast strain was subjected to sequence analysis of the large subunit (LSU) and internal transcribed spacer (ITS) domains of its DNA operon encoding for the ribosomal RNA (rDNA). On the basis of morphological and physiological characteristics, and on the analysis of the two molecular marker sequences, this strain could not be attributed to any known species and it was described as the type strain of *Ogataea uvarum sp. nov.* The first description of the genus *Ogataea* was proposed on the basis of the type species *Ogataea minuta* (Yamada et al., 1994) that showed the assimilation of the potassium nitrate and the presence of asci containing one to four ascospores of pileiform shape. To date, more than 40 species belonging to the genus *Ogataea* have already been described (Péter et al., 2007, 2008; Ji and Bai, 2008; Limtong et al., 2008; Cadez et al., 2013).

The Sanger sequences of the taxonomic markers showed several secondary peaks, raising the question of whether they derived from an internal heterogeneity among the tandem repeat copies of the DNA operon encoding for the rDNA.

In order to analyze separately the rDNA copies, the ITS-LSU sequence was cloned and each clone was sequenced in both directions. This strategy allows for the analysis of single copies and simulates the situation of a metagenomics study based on NGS-based sequencing.

Internal variants of the complete gene cluster and the one of each single gene were registered and compared among themselves and with Sanger sequences of *O. philodendri* and *O. uvarum*. For the first time in our knowledge, this investigation investigated on the variability in the number of the copies of rDNA cistron during the yeast speciation process.

## Materials and Methods

### Grape Sampling and Yeast Isolation

Healthy undamaged Primitivo (*Vitis vinifera*) grape bunches were sampled in a vineyard located at Cutrofiano (Lecce, Southern Italy). Individual grape berries were randomly and aseptically selected from the bunches, to get a 25 g working sample. Epiphytic yeasts were isolated from the sample by washing berries in 250 mL of sterile water on a rotary shaker at 200 rpm for 30 min (Bleve et al., 2006). The sample was centrifuged at 5000 ×*g* for 10 min and the sediment was recovered and suspended in 1 mL of Yeast Peptone Dextrose medium (YPD; Sigma-Aldrich, United States). Sample dilutions from 10^-1^ to 10^-4^ were spread onto YPD agar plates. After incubation at 28°C for 48 h, yeast colonies were submitted to molecular procedures for identification. The morphological and physiological characteristics of the strain were determined by using conventional methods (Yarrow, 1998).

### Enzymatic Activity

Appropriate dilutions of yeast cultures were plated on solid media containing different substrates for the detection of the enzymatic activities. β-glucosidase, amino acid decarboxylase, protease, pectinase, glucanase, and xylanase activity associated with the non-*Saccharomyces* isolates were determined by specific plate assays as previously described (De Benedictis et al., 2011; Tristezza et al., 2012). Hydrogen sulfide production was determined onto Biggy agar plates (Difco, United States) as previously described (Grieco et al., 2011).

Acetic acid and SO_2_ productions were determined as described by Belarbi and Lemaresquier (Belarbi and Lemaresquier, 1994).

### DNA Extraction and Sequencing

The genomic DNA representative of each single morphology was extracted (Bolano et al., 2001; Cardinali et al., 2001). ITS marker was PCR amplified using ITS1 (5′ TCCGTAGGTGAACCTGCGG 3′) and ITS4 (5′ TCCTCCGCTTATTGATATGC 3′) primers (White et al., 1990; Chanchaichaovivat et al., 2007; Tristezza et al., 2013) with the following conditions, denaturation at 94°C for 4 min, 35 cycles at 94°C for 60 s, 48°C for 60 sec and 72°C for 1.5 min, with a final elongation at 72°C for 10 min. The LSU D1/D2 domain was amplified using NL1 (5′-GCATATCAATAAGCGGAGGAAAAG) and NL-4 (5′-GGTCCGTGTTTCAAGACGG) primers (Kurtzman and Robnett, 1998), in the same conditions reported for ITS amplification. PCR products were purified by the PCR Purification Spin Kit (Invitrogen, United States), quantified using NanoDrop^®^ ND-1000 (NanoDrop Technologies, United States) and visualized by gel electrophoresis (Grieco et al., 1999).

The rDNA region (ca. 1400 bp), including ITS1-5.8S rDNA-ITS2-LSU D1/D2 *loci*, was amplified using the ITS1 and NL4 primers (Alves et al., 2005), as follows, an initial denaturation at 96°C for 2.5 min and 25 cycles of 10 s at 96°C, 10 s at 56°C, and 4 min at 60°C. Reactions were run using a PCR Express System (Hybaid, United States). After PCR reactions, the sample were purified and then sequenced by the ABI PRISM 3130 sequencer (Applied Biosystems, United States).

Data outputs were analyzed by the Chromas program version 1.45 and sequences were identified by a database similarity search in the GENBANK Collection using the BLAST^1^ software and CBS^2^ databases.

### PCR Product Cloning and Screening

The PCR products were cloned in pGEM-T Easy vector (Promega) included in pGEM-T Easy Vector System (Promega), following the supplier’s instructions, and ligation reactions (10 μL final volume) were incubated overnight at 4°C. Transformation of *Escherichia coli* DH5α [F- Φ80lacZΔM15 Δ (lacZYA-argF) U169 recA1 endA1 hsdR17 (rK-, mK+) phoA supE44 λ- thi-1 gyrA96 relA1] competent cells was performed and cells were plated onto LB/Ampicillin/IPTG/X-Gal plates (Grieco et al., 1999). Detection of positive clones was performed by colony PCR.

Each reaction (25 μL) contained, Emerald Amp MAX HS PCR Master Mix 2 × Premix (Takara) 12.5 μL, M13 forward primer 0.2 μM, M13 reverse primer 0.2 μM, and sterilized distilled water up to 25 μL. Amplification reactions were performed using the following conditions, 2 min at 98°C (1 hold), 10 s at 98°C, 30 s at 55°C and 45 s at 72°C (25 cycles), followed by a final step of 10 min at 72°C. Plasmid DNA was purified using the Eurogold Plasmid Miniprep Kit I (Euroclone, Italy) and the inserted fragment was sequenced by ABI PRISM 3730xl with primer M13 forward (5′ GTTTTCCCAGTCACGAC 3′) and M13 reverse (5′ CAGGAAACAGCTATGACC 3′). Consensus sequences for each strain and trimming of the ends with low sequencing quality were carried out with Geneious^3^ R6 (v. 6.17, Biomatters, Auckland, New Zealand).

### LSU and ITS Phylogenetic Analysis

Alignment of the ITS and D1/D2 domain of the 26S rDNA (LSU) sequences was carried out in Geneious R6 with Geneious Alignment tool (Bast, 2013). Distances were inferred in MEGA6 (Tamura et al., 2013) the Maximum Composite Likelihood method and expressed as number of base substitutions per site. This procedure has been chosen because it assumes equal substitution patterns and rates among lineages and sites, conditions considered appropriate for a recent and ongoing separation phenomenon. Both transitions and transversions were considered.

The Neighbor-Joining method (Saitou and Nei, 1987) was used to reconstruct the tree with 1000 bootstrap reiterations. Statistical analyses were performed in R environment^4^, on the basis of the genetic distances calculated with MEGA6, as described above.

## Results

### Description of *Ogataea uvarum* Grieco, Corte, Roscini, Cardinali *Sp. Nov.*

**FIGURE 1:**
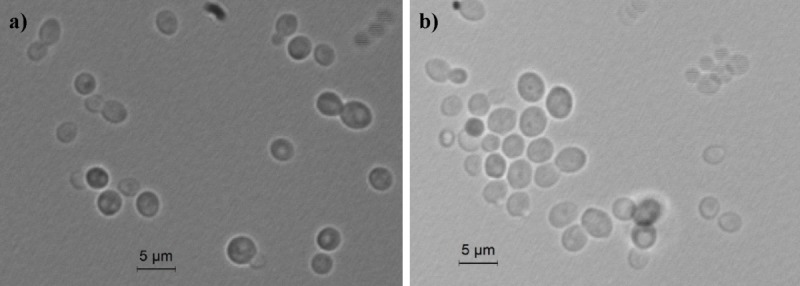
Light microscopic morphology of cells of *O. uvarum* CBS 12829. Panel **(a)** *O. uvarum* cells in YM broth; Panel **(b)** *O. uvarum* cells in YEPD medium.

*Ogataea uvarum* (*u’va’rum, L. n.f.*, pertaining to grapevine, referring to the Latin name of the plant, where the yeast has been isolated the first time). After growth in YM broth at 25°C for 3 days, the cells appeared elliptic shaped (2–4 × 3–5 μm) and occurred singly or in pairs (**Figure 1**).

Vegetative reproduction occurred by multilateral budding. Sediment was not present. After 5 days at 25°C on YM agar, streak cultures showed round colonies with regular edges and a matt white color. On Dalmau slide cultures with corn meal agar or rice extract agar after 5 days at 25°C, pseudomycelium was not formed, neither under the cover glass nor without cover glass. Sporulation did not occur on McClary’s acetate agar, Yeast Extract-Malt Extract (YM) agar at 17°C and 25°C after 10 days. Glucose was not fermented.

D-glucose, [α],[α]-trehalose, glycerol, erythritol, D-mannitol, D-sorbitol, glucosamine, D-xylose, D-ribose, adonitol, L-sorbose, ethanol, 2-keto-D-gluconate, nitrate, glucono-δ-lactone, citric acid, methanol, and lysine were assimilated. Other carbon compounds tested in this study, including, soluble starch, succinic acid, N-acetyl glucosamine, maltose, nitrite, and malic acid were weakly assimilated. D-galctose, sucrose, cellobiose, lactose, melibiose, raffinose, melizitose, inulin, L-arabinose, D-arabinose, L-rhamnose, dulcitol, salicin, DL-lactic acid, inositol, glucuronic acid, α-methyl-D-glucoside, ethylamine, and hexadecane were not assimilated. Growth on 50% glucose and 12.5% NaCl was negative. Growth occurred on 5% NaCl, in presence of 0.1, 1, and 10 ppm cycloheximide and weakly on 10% NaCl. Growth took place at 25, 37, and 42°C but not at 4°C. No starch-like substance was produced. Urea hydrolysis and Diazonium blue B reaction were negative.

Lipase activity was negative. Proteinase activity was weak. Enzyme production assays revealed that this strain was able to decarboxylate histidine and to produce SO_2_ and H_2_S. It showed β-glucosidase activity on arbutin agar. No xylanase activity was detected. Moreover, this strain was able to degrade 1,3-βD-glucan (pachyman) and 1,3,1,4-β-D-glucan (lichenan). Growth carried out on grape-skin and grape-seed agar medium produced dark hazel colonies. Type strain was isolated from grape bunches in a southern Italian region. The culture was deposited in the collection of the Westerdijk Institute, formerly Centraalbureau voor Schimmelcultures (CBS), Utrecht (The Netherlands) as CBS 12829, in the Phaff Yeast Culture as UCDFST 14-401, in the Mycoteque de l‘Universite Catholique de Louvain (MUCL) collection as MUCL 54959. The species description was deposited in the MycoBank database (MB) as MB 810217.

The LSU and ITS rDNA sequences of the new species *O. uvarum* were placed in a well bootstrap supported clade (100%), including members of the genera *Ogataea* and *Candida* globally named *Ogataea* clade (Kurtzman et al., 2011) (Supplementary Figure S1 and Supplementary Table S2). The closest relatives were *Ogataea philodendri* (16 substitutions equivalent to 1.45% difference), *O. polymorpha* (48 substitutions equivalent to 4.36% difference), and *O. angusta* (52 substitutions equivalent to 4.69% difference). Members of the clade rather distant to the new species were *O. kodamae*, known as a species associated with insects (83 substitutions equivalent to 7.55% difference) (Mikata and Yamada, 1995), *O. naganishii*, isolated from plant exudates and rotted logs (94 substitutions equivalent to 8.55% difference) (Kurtzman and Robnett, 2010; Kurtzman et al., 2011), *Candida pignaliae*, usually associated with plants (82 substitutions equivalent to 7.56% difference) (Péter et al., 2010), *O. histrianica* (89 substitutions equivalent to 8.08% difference), *O. kolombanensis* (92 substitutions equivalent to 8.40% difference), and *O. deakii* (108 substitutions equivalent to 9.86% difference), isolated from olive oil and rotten wood (Cadez et al., 2013). The assimilation profile of the proposed species differs for several traits from the closest species of the clade (**Table 1**). In fact, *O. uvarum* assimilates 2-Keto-D-Gluconate, does not assimilate D-ribose, D-xylose, and ribitol, unlike most members of the clade, and does not sporulate like *C. nemodendra* and *C. pignaliae*.

**Table 1:** Comparison of the assimilation profile of selected substrates by species phylogenetically close to *Ogataea uvarum*.

	Species	CBS Number	L-Sorbose	D-Glucosamine	D-Ribose	D-Xylose	Ribitol	2-Keto-D-Gluconate	Succinate	Nitrite	Ethylamine	Glucosamine (N)	at 42°C	spores
***O.***	***uvarum***	**CBS 12829^T^**	**+**	**+**	**-**	**-**	**-**	**+**	**w**	**w**	**-**	**+**	**+**	**-**
*O.*	*philodendri*	CBS 6075^T^	d	**-**	+	+	+	**-**	+	+	+	**-**	**-**	+
*O.*	*minuta*	CBS 1708^T^	**-**	**-**	+	+	+	**-**	**-**, +	nd	nd	Nd	**-**	+
*O.*	*polymorpha*	CBS 4732^T^	v	**-**	+	v	+	**-**	v	+	+	**-**	+	+
*O.*	*nonfermentans*	CBS 5764^T^	**-**		v	v	+	**-**	+	+	+	**-**	nd	+
*O.*	*naganishi*	CBS 6429^T^	**-**	d	+	+	+	**-**	**-**	**-**	+	**-**	**-**	+
*O.*	*angusta*	CBS 7073^T^	d	**-**	+	d	+	**-**	+	+	**-**	**-**	+	+
*O.*	*kodamae*	CBS 7081^T^	**-**, +	**-**	+	+	+	**-**	+	nd	nd	nd	nd	+
*O.*	*dorogensis*	CBS 9260^T^	+	**-**	+	+	+	**-**	+	**-**	nd	nd	nd	+
*C.*	*pignaliae*	CBS 6071^T^	d	**-**	v	+	+	**-**	+	+	+	**-**	**-**	**-**
*C.*	*nemodendra*	CBS 6280^T^	+	**-**	+	+	+	**-**	+	**-**	+	**-**	**-**	**-**

*+ = growth, **-** = no growth, w = weak growth, d = delayed growth, v = variable growth, nd = not determined.*

### The Consensus Sequence Hides the Heterogeneity Among the rDNA Copies

The ITS and LSU D1/D2 sequences of the strain CBS 12829^T^ were obtained with standard procedure after the Sanger sequencing of the respective amplicons.

**FIGURE 2:**
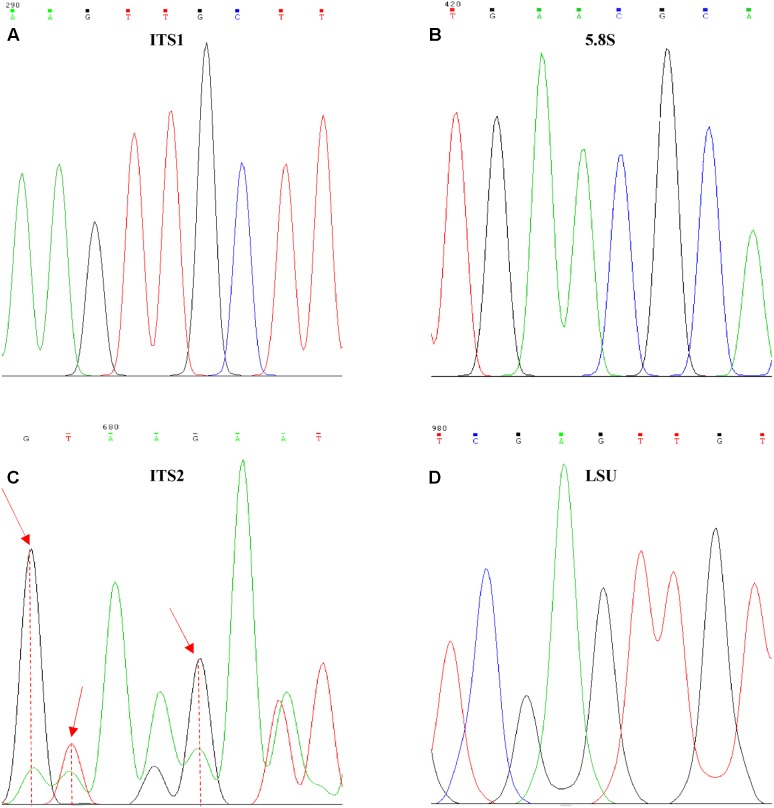
Internal variability on *Ogataea uvarum*^T^ ITS and LSU sequences. Examples of the variability found in the four barcoding genes, **(A)** ITS1, **(B)** 5.8S, **(C)** ITS2, and **(D)** LSU. Red arrows identify positions with high variability degree.

Electropherograms of these “reference sequences” displayed some peaks of lower intensity (hereinafter referred to as secondary peaks) exactly below those of normal height (**Figure 2**), especially in the ITS2 region (**Figure 2C**).

The normal procedure in analyzing Sanger sequencing data is the comparison of forward and reverse strands in order to obtain the consensus, which is considered as the clean “real” sequence. Any uncertainty in this comparison is resolved choosing one of the two possible alternatives, therefore hiding the presence of these internal variants. In some cases, the process is favored by the comparison with the type strain sequence, yielding conservative consensus very similar to that of the type strain, further hiding the level of variation. In order to test the effect of the process of obtaining consensus sequences, distances of ITS and LSU were calculated from both original and consensus sequences. The hypothesis on the effect of the use of a reference sequence was simulated by comparing all the pairwise distances among the clones with those of each strain with its reference.

**FIGURE 3:**
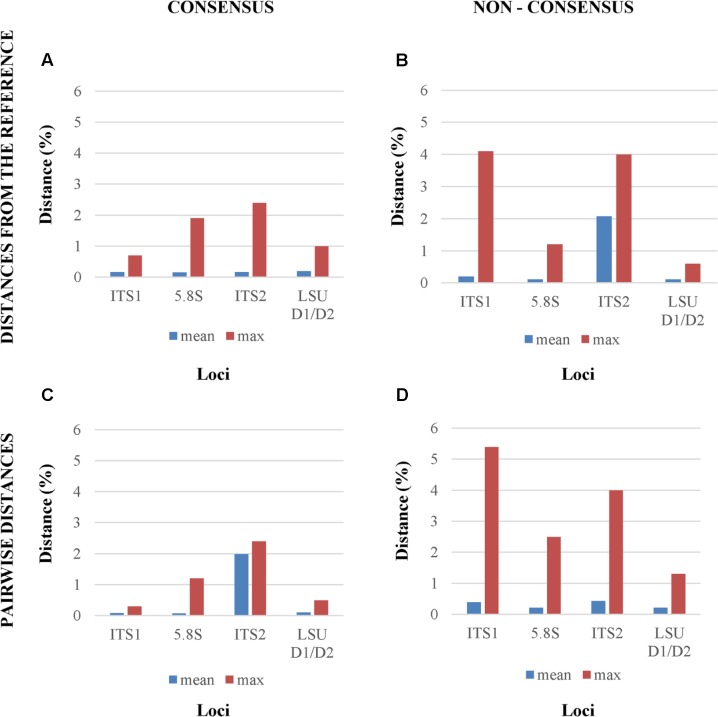
Mean and maximum distances from reference Sanger sequence of four different analytical settings. The four panels report the distances between, **(A)** the reference Sanger Sequence and the cloned consensus sequences; **(B)** the reference Sanger Sequence and the original (non-consensus) cloned sequences; **(C)** and **(D)** all the pairwise distances among the consensus and the original sequences, respectively.

When consensus cloned sequences where compared to the reference, all four tested *loci* showed a relatively low mean distances below 1%, whereas the maximum differences ranged from less than 1% (ITS1) to more than 2% (ITS2), confirming the visual inspection of the electropherograms (**Figure 3A**). The use of non-consensus sequences, hereafter referred to as “original,” produced an increase of mean distances from the reference in all *loci* excluded LSU. The ITS1 and ITS2 maxima reached values close to 4% (**Figure 3B**). When all cloned sequences were compared in a pairwise manner, means and maxima of all *loci* increased (**Figures 3C,D**). Once again, the distances from non-consensus sequences increased more than those obtained with consensus sequences, with maxima spanning from 1 to 5% (**Figure 3D**).

### Independence of the Variations Among the Four *Loci*

Since the rDNA operon is constituted by over 100 tandem repeats in the yeast genome (Dammann et al., 1995) with a degree of variability already studied by different approaches (James et al., 2009; West et al., 2014) these secondary peaks were tentatively attributed to the heterogeneity among repeats. In order to test the relative frequency of variant repeats, the ITS-LSU region was cloned and plasmid borne repeats were sequenced separately in both directions and consensus sequences were obtained. This strategy was chosen to determine the actual frequency of variation among repeats and to test whether a relation exists between the variants in the single *loci* (LSU, ITS1, 5.8S, and ITS2) within the same tandem repeat copy. In order to evaluate the correlation among *loci*, the distance between each clone and the reference sequence was calculated for both consensus and original sequences. The variations among the four *loci* showed independence as indicated by Pearson’s correlation moments close to 0 and very high *p*-values (**Table 2**). Interestingly, the LSU and the 5.8S *loci* were poorly, but negatively correlated in the two conditions studied (**Tables 2A,B**). ITS1 correlated relatively well with the LSU of the consensus (0.381) and with the 5.8S (0.359) of the original sequences, in both cases with an excellent support of the *p*-values. All together, these data support the idea that the variations occurring within the various regions were independent, although some weak pattern has been detected as the negative correlation between the 5.8S and the LSU *loci*.

**Table 2:** Correlation tables among the four *loci* sequences. Lower triangles report the correlations among the distances between the reference sequence and the consensus cloned **(A)** or the original **(B)** sequences.

(A)	ITS1	5.8S	ITS2	LSU
**ITS1**		0.8066	0.6063	0.0088
**5.8S**	0.0371		0.9562	0.3376
**ITS2**	0.0780	0.0090		0.6336
**LSU**	0.3817	**-**0.1446	0.0722	
**(B)**	**ITS1**	**5.8S**	**ITS2**	**LSU**
**ITS1**		0.0007	0.9419	0.1309
**5.8S**	0.3590		0.6699	0.1395
**ITS2**	0.0080	0.0466		0.1375
**LSU**	0.1642	**-**0.1607	0.1614	

*Upper triangles report the p-value of the corresponding correlations.*

### Effects of the rDNA Heterogeneity on the Identification and on the Biodiversity Estimate in a Metagenomics Scenario

The application of Next Generation Sequencing (NGS) technologies to metagenomics has opened effective ways for the determination of the microbial diversity, overcoming several drawbacks bound to the cultivation of microbes. ITS is one of the most used sequences for species identification and has been proposed as universal barcoding marker. LSU was introduced almost two decades ago for species identification and phylogenetic analysis. The application of NGS in metagenomics using these two markers with high copy numbers and high heterogeneity can cause severe over-estimations of the actual diversity. For this reason, an analysis on the distribution of the cloned sequences of the four *loci* was carried out, in order to estimate the effect of these marker sequences in a NGS environment. Values of 1% for LSU (Kurtzman and Robnett, 1998) and 1.59% for ITS (Vu et al., 2016) have been suggested as distance thresholds for the species identification.

**FIGURE 4:**
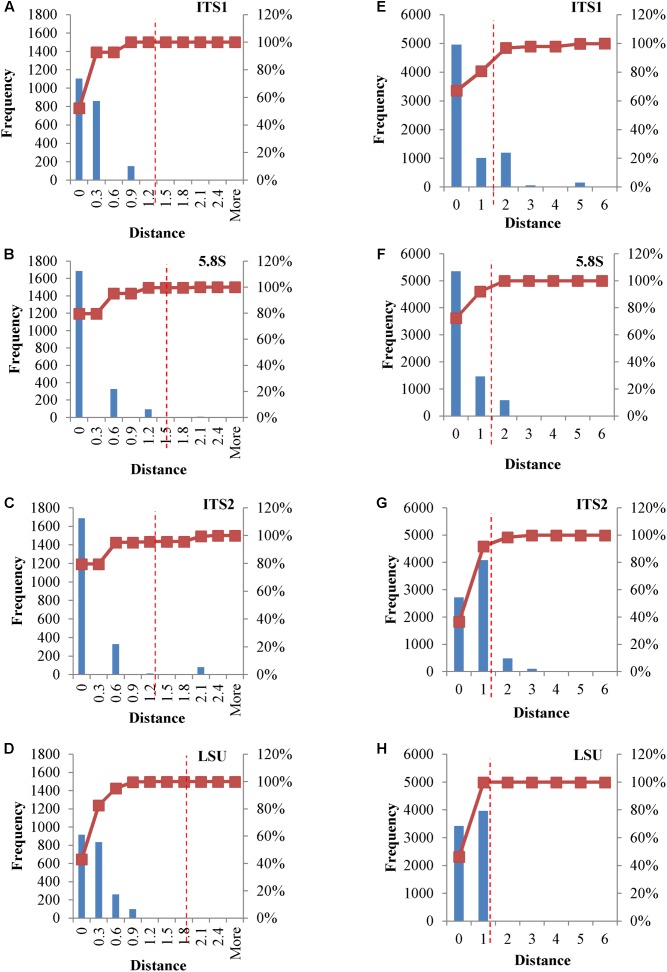
Distance distribution of consensus and original cloned sequences. Panels **(A–D)** Histograms and accumulation curves of the distances between consensus cloned sequences. Panels **(E–H)** Histograms and accumulation curves of the distances between original cloned sequences. Red dotted lines represent the thresholds suggested for species identification.

In metagenomic studies carried out with NGS, single reads could be selected and used for the identification of the species within the sample, thus rising a question on the possible effects caused by the internal heterogeneity of the *loci* selected as species marker. This point was addressed calculating distance matrices of both the original and consensus sequences of the four *loci*, in order to evaluate their distribution. Cloned consensus sequences showed that all the distances fall within the threshold range, with the only exception of 4.25% distances of the ITS2 region (**Figures 4A–D**). When the original sequences were not subjected to the treatment to obtain a consensus, all four *loci* displayed distances extending beyond the threshold limits. Namely, 3.14% of ITS1 and ca 5% of ITS2 distances were larger than 1.59%, whereas these figures were around 1% for LSU and 5.8S (**Figures 4E–H**).

### The Species Differentiation in the View of the SNPs Among rDNA Copies

**FIGURE 5:**
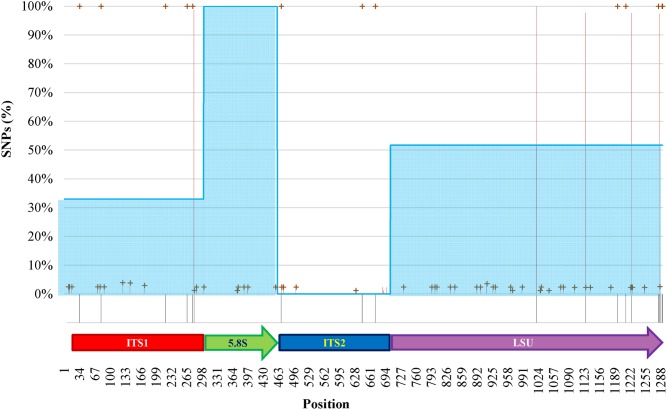
SNPs analysis of clones of *O. uvarum* and *O. philodendri* type strain sequences. Black stripes below the abscissa represent variations between the two type strains. Red lines and green crosses represent the frequency of variants found among the clones when compared to *O. uvarum* and *O. philodendri*, respectively. Cyan areas indicate the average similarity of the clones to the *O. philodendri* type strains in the four loci.

The internal heterogeneity among the rDNA copies produced a series of variants or single nucleotide polymorphisms (SNP). Each variant could be considered a SNP in comparison to the type strain of (1) *O. polymorpha*, (2) *O. uvarum*, (3) both, and (4) none. The ITS1 region showed five differences between the type strains sequences (**Figure 5**). The cloned sequences displayed 8 sites with low frequency variations in comparison to both species Sanger sequences. Five low- and one high-frequency (100%) SNPs were found between the cloned sequences and, respectively, *O. polymorpha* and *O. uvarum*. The 5.8S region displayed only six low frequency SNPs and no difference between the type strains sequences.

In ITS2 three high frequency SNPs were detected only when the clones were compared to *O. polymorpha* type strain sequence, whereas four and five low frequency SNPs were present in comparison with *O. polymorpha* and *O. uvarum*, respectively. Finally, the LSU region displayed 22 low frequency and four high frequency SNPs to *O. uvarum* and 26 low frequency and 5 high frequency SNPs to *O. philodendri*. Most of the low frequency SNPs were present when compared to both species type strains, whereas only two high frequency SNPs were shared by the two type strains sequences. No intermediate frequency SNPs could be detected. High SNPs frequencies were present in all clones, whereas low SNPs frequencies could typically be detected in 2% of the cloned sequences with some cases reaching 4%. Taking the single cloned sequences separately, as would happen in a metagenomics analysis, the number of sequences attributed to *O. polymorpha* would be 32.94% in ITS1, 100% and none in 5.8S and ITS2 and 51.76% in LSU.

**FIGURE 6:**
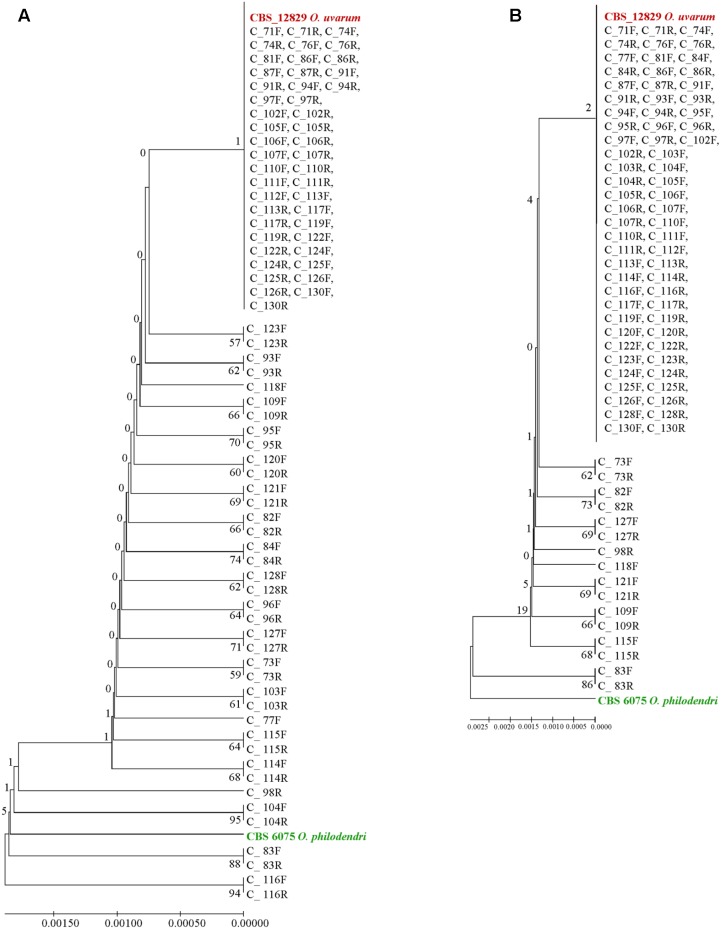
UPGMA phylogenetic trees based on **(A)** concatenated ITS and LSU sequences and **(B)** ITS sequences alone. The evolutionary distances were computed using the Maximum Composite Likelihood method expressed as number of base substitutions per site. UPGMA trees were obtained using the Maximum Composite Likelihood with MEGA6; bootstrap support values were calculated with 100 replicates and are shown next to the branches. GenBank deposit numbers are reported in Supplementary Table S1.

Extending the simulation of a metagenomics study, the concatenated sequences were reported in dendrograms: most of the cloned sequences were classified as *O. uvarum*, while four resulted relatively close to *O. philodendri*, whereas one third occupied an intermediate part of the dendrogram (**Figure 6A**).

When the whole ITS was used, the vast majority of clones were identical to *O. uvarum*, two were close to the other species and 16 were in intermediate positions, although all sequences would fall within the 0.69% species limits recently suggested by Vu et al. (2016) for the ITS barcode (**Figure 6B**).

**FIGURE 7:**
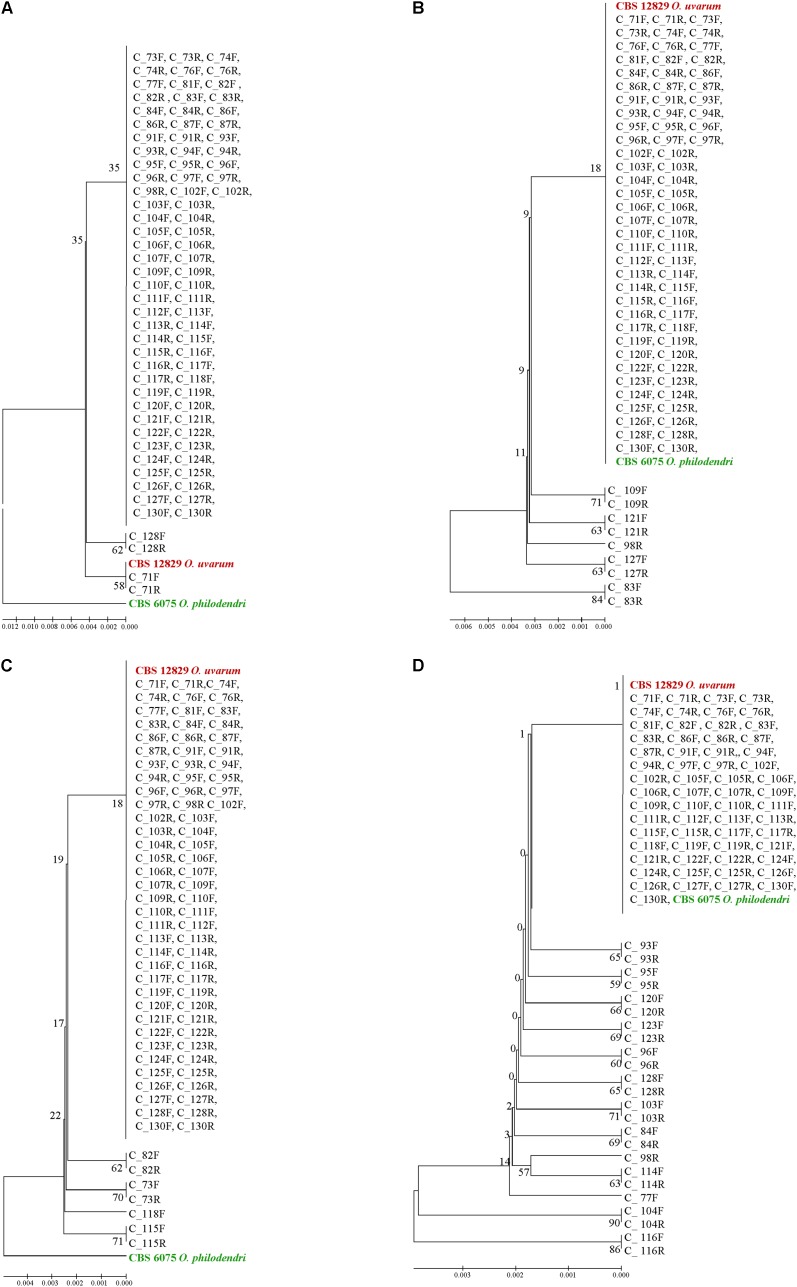
UPGMA phylogenetic trees based on ITS1 **(A)**, 5.8S **(B)**, ITS2 **(C)**, and LSU **(D)** aligned sequences. The evolutionary distances were computed using the Maximum Composite Likelihood method expressed as number of base substitutions per site. UPGMA trees were obtained using the Maximum Composite Likelihood with MEGA6; bootstrap support values were calculated with 100 replicates and are shown next to the branches. GenBank deposit numbers are reported in Supplementary Table S1.

When the ITS1 and ITS2 *loci* were used alone, almost all clones were identical and placed in a large clade close to *O. uvarum* whereas *O. philodendri* resulted well separated (**Figures 7A,C**). With 5.8S, 9 sequences were separated by the large clade containing all other clones and the two type strains (**Figure 7B**). A similar situation was found in LSU with 24 sequences separated from the large group containing all clones and the two type strains (**Figure 7D**). Interestingly, the forward and the reverse sequences of the same clones are normally placed in the same dendrogram leaf, indicating that the variations detected were consistent in the two sequencing directions.

## Discussion

This study relates to a single strain of a newly described species, in which the Sanger sequences of ITS and LSU showed a relatively large number of double peaks, suggesting internal heterogeneity among the copies of the DNA encoding for the ribosomal DNA. The cloning of a sample of single copy sequences showed that indeed the internal heterogeneity is present and that the process of generating a consensus sequence hides a large part of it. This finding is in good agreement with similar results previously obtained with other approaches for other ascomycetous yeasts (James et al., 2009; West et al., 2014). The heterogeneous *loci* of interest are the ITS1 and ITS2, whereas the LSU and the 5.8S displayed a moderate amount of variability. Cloning the whole region spanning from ITS1 to LSU D1/D2 allowed to compare the level of variability of the single *loci* within each clone, showing a low degree of correlation that suggesting that the variations occur independently among the single *loci* within the same copy.

The impact of NGS in metagenomics studies allows to hypothesize that these *loci* could be increasingly used to describe the species present in the samples and the extent of alpha- diversity, as also indicated by a recent study on sequence variation in the ITS and LSU regions of *Rizhophagus irregularis* and *Gigaspora margarita* (Thiéry et al., 2016). According to our data and analysis, the internal heterogeneity LSU D1/D2 *locus* is moderate and therefore does not create problems in the alpha biodiversity estimate. On the other hand, the variability among the repeats of the ITS is much larger and is likely to produce more serious overestimates of diversity and misidentifications, as demonstrated in a recent study carried out with different yeast species (Colabella et al., 2018).

As long as fungal taxonomic descriptions will be restricted to isolated strains, this internal heterogeneity will be not expected to produce problems of misidentification, neither with Sanger, nor with NGS sequencing. In fact, the former requires a thorough cleaning process that purges the consensus sequences from most if not all the effects of the variants. On the other hand, if NGS is applied as an alternative to Sanger to sequence single strain *loci*, the heterogeneity is expected to be displayed but, once again, purged by the process of generating a consensus. However, an internal heterogeneity will cause problems of misidentification when the NGS approach is employed within metagenomics strategies to explore the undescribed fungal diversity, maybe accounting for some 98% of the total or even more (Hawksworth and Rossman, 1997). Whether other species would show the same extent of the problem or will exhibit different figures is an issue requiring further investigation with more strains and species. For the current understanding, the internal heterogeneity is a sort of noise within otherwise quite similar copies of the rDNA genes. The fact that diverse species show significantly different sequences of both LSU (Kurtzman and Robnett, 1998) and ITS (Schoch et al., 2012) led to use them as tools in taxonomy and barcoding. The question on how the various copies change more or less simultaneously in a newly formed species has been long debated and mechanisms spanning from the concerted evolution to the birth-and-death evolution have been considered (Nei and Rooney, 2005). The different rate of variation found in the two *loci* encoding the rRNA vs. the two ITSs suggests that some sort of purging selection occurs, regardless of the concerted evolution mechanism. In fact, low frequency variants are particularly common in the two rDNA encoding *loci*, suggesting that they are not eliminated by concerted evolution. This suggests that maybe concerted evolution is exerted more efficiently against high frequency variants, particularly common in the non-coding regions. This mechanism implies that some variants remain somehow conserved even after the speciation event, as shown by the consistent presence of internal heterogeneity among yeast (West et al., 2014).

All variations between the two type strains corresponded to high frequency SNPs, mostly to *O. philodendri*, four to *O. uvarum* and two to both. These evidences suggest that the direct Sanger sequencing of an amplicon records only the high frequency variants, whereas the other are maybe visible as double peaks.

The results here reported indicated that the internal heterogeneity of the rDNA operon must be taken into account when this genomic region is used as DNA barcode in NGS-based metagenomic studies aiming at biodiversity estimation and identification of mixed microbial populations. For the first time in our knowledge, this investigation investigated on the variability in the number of the copies of rDNA cistron during the yeast speciation process.

However, since the species separation is largely based on the two multi-copy markers ITS (Schoch et al., 2012) and LSU (Kurtzman and Robnett, 1998), a further understanding of these mechanisms seems to be advisable for the correct use of these markers. On the other hand, the possibility that the low copy variants differ among strains, could be employed for their characterization.

## Author Contributions

LR performed physiological characterization and contributed to bioinformatics analysis of sequencing data and to generation to the figures. MT isolated the new species and performed its preliminary characterization. LC contributed to the writing of the manuscript and led the final editing prior to the submission. CC performed DNA extraction and PCR amplification for “Reference Sequences.” CP and PR performed the amplification and cloning experiments. VR and DV contributed to perform bioinformatics analysis. GC contributed to conceive and design the experiments, contributed to perform bioinformatics and statistical analysis, led the writing of the manuscript and the generation of the figures. FG contributed to conceive and design the experiments, to isolate the new species, and to perform its preliminary characterization, to the writing of the manuscript. All authors read and approved the final submitted version of the manuscript.

## Conflict of Interest Statement

The authors declare that the research was conducted in the absence of any commercial or financial relationships that could be construed as a potential conflict of interest.

## Footnotes

1 http://www.ncbi.nlm.nih.gov/BLAST/

2 http://westerdijkinstitute.nl

3 www.geneious.com

4 http://www.r-project.org
