# Supplementary material for: Early Ongoing Speciation of Ogataea uvarum Sp. Nov. Within the Grape Ecosystem Revealed by the Internal Variability Among the rDNA Operon Repeats
Source: Front Microbiol. 2018 Aug 3;9:1687. doi: 10.3389/fmicb.2018.01687 (PMC6085423; doi:10.3389/fmicb.2018.01687)
Supplement: Supplementary file 1 [file Data_Sheet_1.pdf]

Supplementary Material

Early ongoing speciation of *Ogataea uvarum Sp. Nov.* within the grape ecosystem revealed by the internal variability among the rDNA operon repeats

Luca Roscini[1◆], Mariana Tristezza [2◆], Laura Corte[1], Claudia Colabella[1], Carla Perrotta[3], Patrizia Rampino[3], Vincent Robert[4], Duong Vu[4], Gianluigi Cardinali[1,5]* and Francesco Grieco[2]*

[1] *Department of Pharmaceutical Sciences - Microbiology, University of Perugia, Perugia, Italy,* [2] *Institute of Sciences of Food Production (ISPA), National Research Council (CNR), Lecce (Italy),* [3] *Department of Biological and Environmental Sciences and Technologies, University of Salento, Lecce, Italy,* [4] *Bioinformatics Unit – Westerdijk Fungal Biodiversity Institute – Utrecht, Netherlands,* [5] *CEMIN, Centre of Excellence on Nanostructured Innovative Materials, Department of Chemistry Biology and Biotechnology, University of Perugia, Perugia, Italy.*

◆ *Both authors contributed equally to this work*

***CORRESPONDENCE**

Dr. Gianluigi Cardinali

gianluigi.cardinali@unipg.it

Dr. Francesco Grieco

e.mail, francesco.grieco@ispa.cnr.it

Table S1. GenBank deposit numbers of the sequences of each *O. uvarum* clone obtained by using the M13 forward and M13 reverse primers and the consequent consensus sequences are shown.

SampleID	GenBank Number	Sequence Type
C_71	KY971642	Consensus
C_71_F	KY930781	Forward
C_71_R	KY930782	Reverse
C_73	KY971643	Consensus
C_73_F	KY930783	Forward
C_73_R	KY930784	Reverse
C_74	KY971644	Consensus

C_74_F	KY930785	Forward
C_74_R	KY930786	Reverse
C_76	KY971645	Consensus
C_76_F	KY930787	Forward
C_76_R	KY930788	Reverse
C_77_F	KY930789	Forward
C_81_F	KY930790	Forward
C_82	KY971646	Consensus
C_82_F	KY930791	Forward
C_82_R	KY930792	Reverse
C_83	KY971647	Consensus
C_83_F	KY930793	Forward
C_83_R	KY930794	Reverse
C_84	KY971648	Consensus
C_84_F	KY930795	Forward
C_84_R	KY930796	Reverse
C_86	KY971649	Consensus
C_86_F	KY930797	Forward
C_86_R	KY930798	Reverse
C_87	KY971650	Consensus
C_87_F	KY930799	Forward
C_87_R	KY930800	Reverse
C_91	KY971651	Consensus
C_91_F	KY930801	Forward
C_91_R	KY930802	Reverse
C_93	KY971652	Consensus
C_93_F	KY930803	Forward
C_93_R	KY930804	Reverse
C_94	KY971653	Consensus
C_94_F	KY930805	Forward
C_94_R	KY930806	Reverse
C_95	KY971654	Consensus
C_95_F	KY930807	Forward
C_95_R	KY930808	Reverse
C_96	KY971655	Consensus

C_96_F	KY930809	Forward
C_96_R	KY930810	Reverse
C_97	KY971656	Consensus
C_97_F	KY930811	Forward
C_97_R	KY930812	Reverse
C_98_R	KY930813	Reverse
C_102	KY971658	Consensus
C_102_F	KY930814	Forward
C_102_R	KY930815	Reverse
C_103	KY971659	Consensus
C_103_F	KY930816	Forward
C_103_R	KY930817	Reverse
C_104	KY971660	Consensus
C_104_F	KY930818	Forward
C_104_R	KY930819	Reverse
C_105	KY971661	Consensus
C_105_F	KY930820	Forward
C_105_R	KY930821	Reverse
C_106	KY971662	Consensus
C_106_F	KY930822	Forward
C_106_R	KY930823	Reverse
C_107	KY971663	Consensus
C_107_F	KY930824	Forward
C_107_R	KY930825	Reverse
C_109	KY971664	Consensus
C_109_F	KY930826	Forward
C_109_R	KY930827	Reverse
C_110	KY971665	Consensus
C_110_F	KY930828	Forward
C_110_R	KY930829	Reverse
C_111	KY971666	Consensus
C_111_F	KY930830	Forward
C_111_R	KY930831	Reverse
C_112_F	KY930832	Forward
C_113	KY971668	Consensus
C_113_F	KY930833	Forward

C_113_R	KY930834	Reverse
C_114	KY971669	Consensus
C_114_F	KY930835	Forward
C_114_R	KY930836	Reverse
C_115	KY971670	Consensus
C_115_F	KY930837	Forward
C_115_R	KY930838	Reverse
C_116	KY971671	Consensus
C_116_F	KY930839	Forward
C_116_R	KY930840	Reverse
C_117	KY971672	Consensus
C_117_F	KY930841	Forward
C_117_R	KY930842	Reverse
C_118_F	KY930843	Forward
C_119	KY971674	Consensus
C_119_F	KY930844	Forward
C_119_R	KY930845	Reverse
C_120	KY971675	Consensus
C_120_R	KY930846	Reverse
C_120_F	KY930847	Forward
C_121	KY971676	Consensus
C_121_F	KY930848	Forward
C_121_R	KY930849	Reverse
C_122	KY971677	Consensus
C_122_F	KY930850	Forward
C_122_R	KY930851	Reverse
C_123	KY971678	Consensus
C_123_F	KY930852	Forward
C_123_R	KY930853	Reverse
C_124	KY971679	Consensus
C_124_F	KY930854	Forward
C_124_R	KY930855	Reverse
C_125	KY971680	Consensus
C_125_F	KY930856	Forward
C_125_R	KY930857	Reverse

C_126	KY971681	Consensus
C_126_F	KY930858	Forward
C_126_R	KY930859	Reverse
C_127	KY971682	Consensus
C_127_F	KY930860	Forward
C_127_R	KY930861	Reverse
C_128	KY971683	Consensus
C_128_F	KY930862	Forward
C_128_R	KY930863	Reverse
C_130	KY971684	Consensus
C_130_F	KY930864	Forward
C_130_R	KY930865	Reverse

Table S2. GenBank deposit numbers of the taxa related to *Ogatea uvarum*.

Species	***Strain n.***	***ITS***	***LSU***
Ogataea uvarum	CBS 12829	HE965024	LN849460
Candida nemodendra	CBS 6280	CBS 6280 ex 10202 ITS	U70246
Ogataea naganishii	CBS 6429	cr - CBS 6429	U75724
Ogataea pignaliae	CBS 6071	cr - CBS 6071	U70183
Ogataea philodendri	CBS 6075	cr - CBS 6075	CBS 6075 ex 46790 116657 LSU
Ogataea polymorpha	CBS 4732	FJ914915	FJ914932
Ogataea angusta	CBS 7073	JF756588	FJ914931
Ogataea nonfermentans	CBS 5764	cr - CBS 5764	U75518
Ogataea kodamae	CBS 7081	cr - CBS 7081	U75525
Ogataea dorogensis	CBS 9260	CBS 9260 ex 21244 6403 ITS	AF403146
Ogataea minuta	CBS 1708	*na*	U75515
Ogataea histrianica	CBS 12779	HE799677	HE799677-2
Ogataea kolombanensis	CBS 12778	HF559222	FR690079
Ogataea deakii	CBS 12375	KC252610	GQ265921

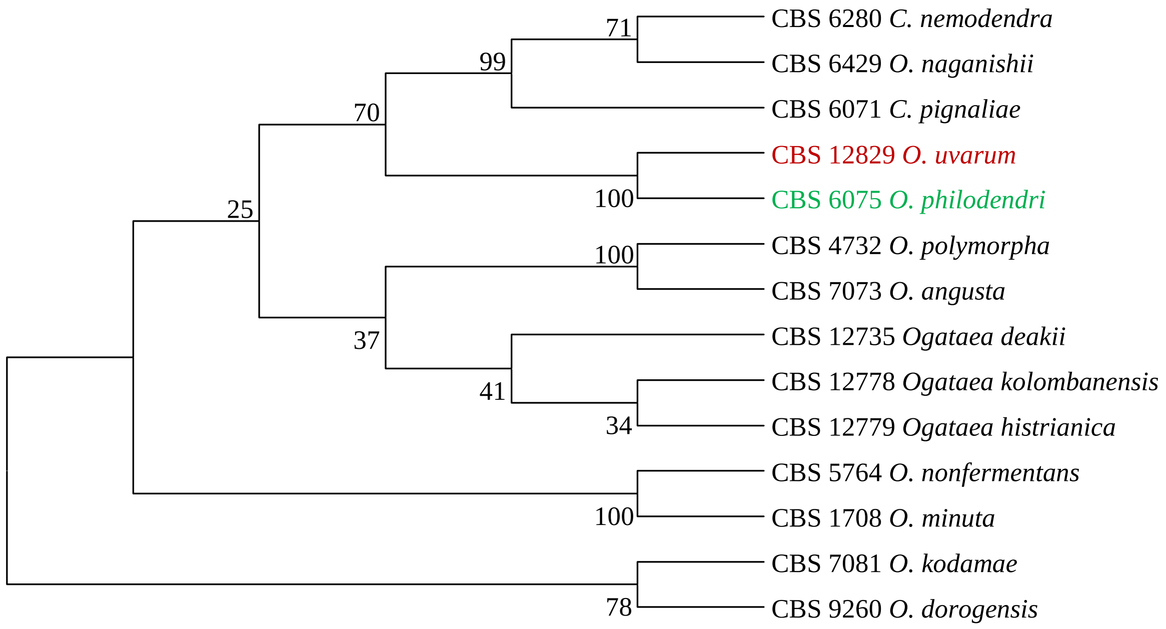

Supplementary Fig. 1 Evolutionary relationships of 13 taxa related to *O. uvarum*. Phylogenetic tree reconstructed using the Maximum Parsimony (MP) method on previously aligned and concatenated ITS and LSU sequences. The optimal tree is shown, with bootstrap support (1000 replicates) shown next to the branches. The MP tree was obtained using the Subtree-Pruning-Regrafting (SPR) algorithm in MEGA6. Type strains sequences were retrieved from GenBank and CBS databases. GenBank deposit numbers are reported in Table S2.
